# Multiplex PCR-Lateral Flow Dipstick Method for Detection of Thermostable Direct Hemolysin (*TDH*) Producing *V. parahaemolyticus*

**DOI:** 10.3390/bios13070698

**Published:** 2023-06-30

**Authors:** Jirakrit Saetang, Phutthipong Sukkapat, Suriya Palamae, Prashant Singh, Deep Nithun Senathipathi, Jirayu Buatong, Soottawat Benjakul

**Affiliations:** 1 International Center of Excellence in Seafood Science and Innovation, Faculty of Agro-Industry, Prince of Songkla University, Hat Yai 90110, Songkhla, Thailand; jirakrit.s@psu.ac.th (J.S.); phutthipong.su@psu.ac.th (P.S.); suriya.pal@psu.ac.th (S.P.);; 2 Department of Nutrition, and Integrative Physiology, Florida State University, Tallahassee, FL 32306, USA; psingh2@fsu.edu

**Keywords:** *V. parahaemolyticus*, PCR, lateral flow dipstick, seafood, *TLH*, *TDH*

## Abstract

*Vibrio parahaemolyticus* is usually found in seafood and causes acute gastroenteritis in humans. Therefore, a detection method of pathogenic *V. parahaemolyticus* is necessary. Multiplex PCR combined with lateral flow dipstick (LFD) assay was developed to detect pathogenic *V. parahaemolyticus*. Biotin-, FAM-, and Dig-conjugated primers targeting thermolabile hemolysin (*TLH*) and thermostable direct hemolysin (*TDH*) genes were used for multiplex PCR amplification. The condition of the method was optimized and evaluated by agarose gel electrophoresis and universal lateral flow dipstick. The specificity assay was evaluated using strains belonging to seven foodborne pathogen species. The sensitivity of the method was also evaluated using DNA in the concentration range of 0.39–100 ng/reaction. The artificial spiking experiment was performed using 10 g of shrimp samples with an enrichment time of 0, 4, and 8 h with 10^1^, 10^2^, and 10^3^ CFU of *V. parahaemolyticus*. The developed multiplex PCR-LFD assay showed no non-specific amplification with a limit of the detection of 0.78 ng DNA/reaction visualized by agarose gel electrophoresis and 0.39 ng DNA with LFD assay. The artificial spiking experiment demonstrated that this method could detect pathogenic *V. parahaemolyticus* at 10 CFU/10 g shrimp samples following a 4 h of enrichment. Multiplex PCR-LFD assay was therefore established for detecting pathogenic *V. parahaemolyticus* with high sensitivity and specificity and might be a useful tool to develop a detection kit used in the food safety sector.

## 1. Introduction

*V. parahaemolyticus* is a Gram-negative, halophilic marine bacterium that contributes to seafood-related gastroenteritis through the consumption of contaminated raw or undercooked seafood [1]. Normally, 10^5^ to 10^7^ cells are considered the infective dose for disease pathogenicity, which has an incubation period of 4–96 h, resulting in an illness that lasts for 2–3 days [2]. Although the infection of *V. parahaemolyticus* is normally self-recovering, the outbreaks can negatively cause economic loss for the seafood industry, particularly marine aquaculture farms, seafood markets, and restaurants [3]. The *V. parahaemolyticus* strains are typically found in brackish water and grow very well in warm conditions, where the temperature is higher than 15 °C and the concentration of sodium chloride is lower than 25 ppm [4]. The *V. parahaemolyticus* strains have a doubling time of around 8–9 min, which enables their rapid spread and proliferation. Since *V. parahaemolyticus* strains are an inhabitant of the natural marine ecosystem, a strong surveillance program that tests for pathogenic *V. parahaemolyticus* strains would be more effective.

Patients infected by *V. parahaemolyticus* commonly display certain clinical symptoms, such as diarrhea, abdominal cramps, low-grade fever, and vomiting [5]. Various virulent factors are responsible for the pathogenesis of these clinical symptoms, including thermostable direct hemolysin (*TDH*), *TDH*-related hemolysin (*TRH*), type III secretion systems (i.e., T3SS1 and T3SS2), lipopolysaccharide, and extracellular proteases [6]. Several studies have demonstrated that most clinical strains of *V. parahaemolyticus* (>90%) possess the *TDH* and/or *TRH* genes in their genome while these genes are rarely found in the environmental isolates (<1%) [7]. *TDH* is a toxin gene that mediates the formation of the pores on erythrocyte cell membrane and activates the ion channels. Those phenomena lead to the leakage of water and ions from the cell and the increase of ion flux in the gastrointestinal tract, responsible for the diarrhea during infection [6]. *TRH* is another toxin gene that shares 70% similarity of the sequence to *TDH*. The mechanism of the pathogenicity and immunological activity of this gene is similar to that of *TDH*, but it is heat labile [6]. Multiple epidemiological investigations have reported a high proportion of pathogenic *V. parahaemolyticus* isolated from patients in southeast Asian countries which always display the *TDH*^+^ genotype [8,9,10,11]. 

Monitoring and detection of pathogenic *V. parahaemolyticus* strains in seafood specimens are important for food safety and surveillance. Therefore, diagnostic assays that can specifically identify pathogenic *V. parahaemolyticus* strains (*TDH*^+^) from the non-pathogenic environmental strains are needed for enhancing food safety and *V. parahaemolyticus*-associated cases. Basically, thermolabile hemolysis gene (*TLH*) is usually employed for *V. parahaemolyticus* detection. *TLH* functions as an enzyme phospholipase, and its hemolysis activity can be found only in the presence of lecithin [6]. This gene was observed in both clinical and environmental isolates. Thus, it has been employed as a target for the development of the *V. parahaemolyticus* detection method [6]. 

Conventional culture methods for the detection and identification of pathogenic *V. parahaemolyticus* in foods are laborious and time-consuming. The multistep procedures involve food matrix preparation, enrichment, screening, microscopic observation, and biochemical testing [5]. Further, these culture-based methods are prone to human error and can result in false-positive or false-negative data interpretation [5]. Nowadays, many types of biosensors have been developed for food safety assessment, especially electrochemical sensors and DNA-immunobiosensors [12,13]. Polymerase chain reaction (PCR) method and its extended modification have been widely used to identify pathogenic *V. parahaemolyticus* from clinical, environmental, and seafood samples [14]. These PCR-based methods are sensitive and reliable for the detection and differentiation of pathogenic *Vibrio* spp. from non-pathogenic environmental strains using the combination of primers targeting different virulence genes or species [14,15]. However, the visualization of the result of conventional PCR requires an agarose gel electrophoresis step with staining and destaining of DNA binding dye, making the whole detection a 24–48 h long process [16]. Although the real-time PCR technique has been developed to monitor the amplification during the runtime, the instrument is quite expensive and is consequently not applicable to all laboratories [17]. To tackle this problem and simplify the diagnostic process, a lateral flow dipstick (LFD) assay has been developed and combined with the DNA-immunobiosensor assay [17]. This method requires only 10–15 min to perform, whereas agarose gel electrophoresis needs 45–50 min to complete the visualization [18]. 

The present study aimed to develop the rapid multiplex PCR lateral flow dipstick (PCR-LFD) immunobiosensor assay for the specific detection of pathogenic *V. parahaemolyticus* strains. For this assay, the thermolabile hemolysis gene (*TLH*) was selected as this marker is observed in all *V. parahaemolyticus* strains [19]. Additionally, the *TDH* gene marker was used as a pathogenic *V. parahaemolyticus* marker as more than 80% of clinical strains of *V. parahaemolyticus* contain this toxin gene in their genome [20]. This is the first study that combined multiplex PCR and LFD assay to identify and differentiate pathogenic *V. parahaemolyticus* from non-pathogenic strains by using *TLH* and *TDH* genes.

## 2. Materials and Methods

### 2.1. Bacterial Strains, Culture Medium, and Spiked Sample Preparation

The clinical strains of *V. parahaemolyticus* (*TDH*^+^*TRH*^−^*TLH*^+^) were isolated from patients at Songklanagarind Hospital, Faculty of Medicine, Prince of Songkla University (Songkhla, Thailand). The environmental strains of *V. parahaemolyticus* (*TDH*^−^*TRH*^−^*TLH*^+^) were isolated from Asian Green mussels (*Perna viridis*) and were used as a non-pathogenic strain. All strains of *V. parahaemolyticus* were identified and confirmed for their species, genotypes, and phenotypes using standard culture and molecular procedures described in Chapter 9 of the Bacteriological Analytical Manual (BAM) protocol [21]. *Escherichia coli* ATCC 25922, *Staphylococcus aureus* ATCC 25923, *Pseudomonas aeruginosa* ATCC 27853, *Listeria monocytogenes* ATCC 15313 were obtained from the American Type Culture Collection (ATCC, Manassas, VA, USA). *Shewanella putrefaciens* JCM 20190 was obtained from the Japan Collection of Microorganisms (RIKEN BioResource Research Center, Ibaraki, Japan). *Shewanella* sp. TBRC 5775 was donated by Thailand National Center for Genetic Engineering and Biotechnology (National Science and Technology Development Agency (NSTDA), Pathum Thani, Thailand). *Shigella sonnei* PSU.SCB.16S.14 was a gift from the Food Safety Laboratory, Prince of Songkla University (Songkhla, Thailand).

All non-*V. parahaemolyticus* species, except *L. monocytogenes*, were cultured on tryptic soy agar and/or tryptic soy broth (Merck Millipore, Darmstadt, Germany) at 35–37 °C for 16–18 h. Brain heart infusion agar and/or broth (Merck Millipore, Darmstadt, Germany) were used for *L. monocytogenes* culture. *V. parahaemolyticus* strains were grown on tryptic soy agar and/or broth supplemented with 3% sodium chloride and cultured at 37 °C for 16–18 h. 

Spiked shrimp samples were prepared and inoculated following the previously described procedure with a few modifications [21]. Briefly, frozen Pacific white shrimp (*Litopenaeus vannamei*) were purchased from the local market of Hat Yai (Songkhla, Thailand) and tested for the absence of pathogenic *V. parahaemolyticus* strains using conventional culture test and PCR assay [22]. 

### 2.2. DNA Extraction

The genomic DNA of *V. parahaemolyticus* and non-*V. parahaemolyticus* strains were extracted using a QIAamp DNA Mini Kit (Qiagen, Hilden, Germany) according to the manufacturer’s instructions. Briefly, microbial cell pellets were collected from 3 mL of overnight pure culture and lysed in 200 µL of lysis buffer containing 20 µL of protease K supplied with the kit. Samples were incubated at 56 °C for 1 h before adding buffer AL (200 µL) and absolute ethanol (200 µL). The mixture was added to the DNeasy Mini spin column followed by washing using buffer AW1 and AW2, two times each. Finally, extracted DNA was eluted with 50 µL of buffer AE followed by the measurement for quality and quantity using NanoDrop™ Lite Spectrophotometer (Thermo Fisher, Waltham, MA, USA). All DNA samples were stored at −20 °C until further experiment was carried out. 

### 2.3. PCR and PCR-LFD Assay

Both PCR and multiplex PCR reactions were carried out using an AllTaq master mix kit (Qiagen, Hilden, Germany) with a total volume of 20 µL. The sequences of *TDH* and *TLH* primer pairs are shown in Table 1, and these pairs of primers were used separately in the conventional PCR reaction. All amplified reactions, excluding sensitivity test reactions, were completed with 50 ng/reaction of the DNA isolated from pathogenic *V. parahaemolyticus* with the final concentration of 0.4 mM of each nucleotide primer. The PCR condition was started at 95 °C for 2 min for pre-denaturation followed by 30 cycles of (95 °C for 5 s, 60 °C for 15 s, and 72 °C for 10 s) before ending the reaction with a final extension step at 72 °C for 10 min, and samples at the end were maintained at 4 °C. For multiplex PCR setup, both pairs of primers were used in the same reaction with a similar condition of amplification as described above. All PCR reactions were performed with the Eppendorf™ Mastercycler™ Nexus Thermal Cycler (Eppendorf, Hamburg, Germany). The amplicons were visualized by using 2% agarose gel electrophoresis stained with 1 µg/mL ethidium bromide.

**Figure 1:**
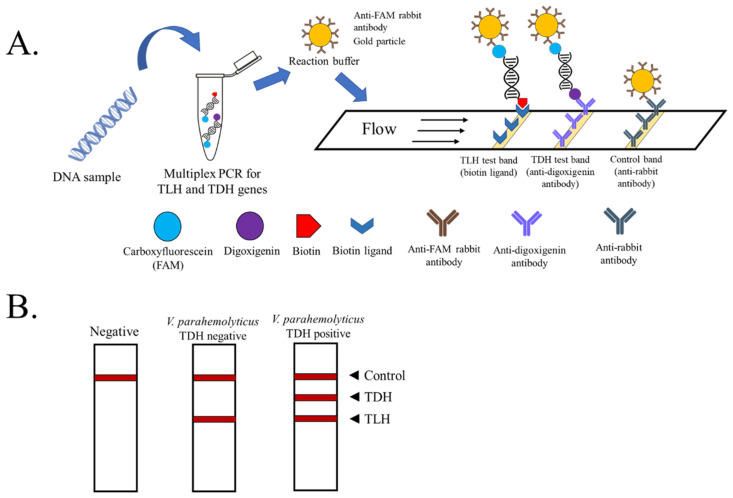
Schematic representation of the PCR product visualized by lateral flow assay (test strip from Milenia Biotec). (**A**) The principle of multiplex PCR-LFD for the detection and differentiation of pathogenic *V. parahaemolyticus*. (**B**) In the absence of amplicons of *TLH* and *TDH*, color appears at a control line only. If only *TLH* amplicon is found, the environmental strain of *V. parahaemolyticus* is presumed. The appearance of all three lines on the strip indicates the presence of pathogenic *TDH*^+^ *V. parahaemolyticus* in the sample.

For the LFD assay, HybriDetect 2T universal lateral flow dipstick (Milenia Biotec, Giessen, Germany) was used. After the PCR amplification, 10 µL of the product was mixed with 100 µL HybriDetect Assay Buffer and vortexed before the dipstick was placed into the mixture. The strip was incubated for 5–10 min, in which a positive result was recorded based on the lines appearing on the test strips. The result is based on the specificity between anti-FAM antibodies coated on nanogold particles and FAM-conjugated PCR products and the trapping of amplicon-anti-FAM antibodies complex on the strip, which relies on the presence of biotin and digoxigenin on *TLH* and *TDH* amplicon, respectively (Figure 1A). After applying the mixture between multiplex PCR amplicon and LFD assay buffer to the strip, the visible red bands are observed when the complex is bound by biotin ligand (test line 1) and/or anti-digoxigenin antibodies (test line 2). The free anti-FAM antibodies coated on nanogold particles will be bound to the anti-rabbit antibodies at the control line, indicating that no reaction of the error happens. For pathogenic *V. parahaemolyticus*, both *TLH* and *TDH* were amplified while only *TLH* was amplified for non-pathogenic *V. parahaemolyticus* DNA samples. The positive result is found at test line 1 and the control line, denoting the presence of *V. parahaemolyticus* environmental strains in the sample (Figure 1B). When only the control line is visible, no *V. parahaemolyticus* is found in the sample (Figure 1B). 

### 2.4. Specificity and Sensitivity Testing

Specificity of the multiplex PCR reaction was performed toward DNA isolated from seven species of non-*V. parahaemolyticus* pathogenic bacteria, including *Escherichia coli* ATCC 25922, *Staphylococcus aureus* ATCC 25923, *Pseudomonas aeruginosa* ATCC 27853, *Listeria monocytogenes* ATCC 15313, *Shewanella putrefaciens* JCM 20190, *Shewanella* sp. TBRC 5775, and *Shigella sonnei* PSU.SCB.16S.14. A sensitivity test was performed using the different amounts of DNA extracted from *V. parahaemolyticus* clinical strain for multiplex PCR reactions, including 100, 50, 25, 12.5, 6.25, 3.12, 1.56, 0.78, and 0.39 ng/reaction. The amplicons from both the specificity test and sensitivity test were visualized using 2% agarose gel electrophoresis stained with 1 µg/mL ethidium bromide and/or LFD assay.

### 2.5. Artificial Spiking Experiment

Ten grams of the middle part of the frozen shrimp was ground using a sterile blade before the addition of 10^1^, 10^2^, and 10^3^ CFU of *TDH*^+^ pathogenic *V. parahaemolyticus* strain PSU.HVP1. The prepared paste was submerged in 100 mL of alkaline peptone water (APW) (polypeptone, 10 g/L; NaCl, 20 g/L; pH 8.6). The mixture was incubated in a shaker incubator at 37 °C, 150 rmp, and 1 mL enrichment was sampled at 0, 4, and 8 h time intervals. The cell pellet was harvested by centrifugation at 10,621× *g* for 1 min (Eppendorf 5430R centrifuge, Hamburg, Germany). The obtained cell pellets were used for DNA extraction using QIAamp DNA Mini Kit. Fifty nanograms of DNA was used as a starting template for the multiplex-PCR-LFD experiment.

## 3. Results

### 3.1. Optimization of Multiplex PCR-LFD Assay

**Figure 2:**
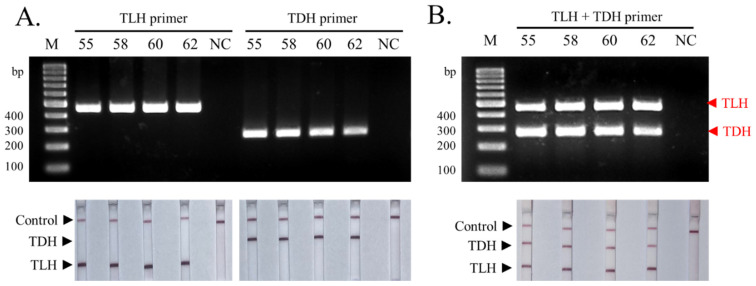
Optimization of the annealing temperature for *TLH* and *TDH* primers using a DNA sample extracted from a pure culture of pathogenic *V. parahaemolyticus*. (**A**) The result of each annealing temperature on single primer PCR was visualized by agarose gel electrophoresis and LFD assay. (**B**) The result of each annealing temperature on multiplex PCR was visualized by agarose gel electrophoresis and LFD assay. The numbers 55, 58, 60, and 62 indicate the temperatures used for annealing. Lane M: 100 bp DNA marker. NC: negative control.

The optimal conditions of each single primer pair were determined by testing primers at multiple annealing temperatures between 55 °C and 62 °C. The results showed that all 55, 58, 60, and 62 °C annealing temperatures generated a strong *TLH* gene amplification product (Figure 2A upper panel) without any non-specific amplification. Similarly, the *TDH* primer generated a specific PCR band in between 55–60 °C annealing temperatures, and a weak intensity band at 62 °C. The LFD assays were performed for all the abovementioned *TLH* and *TDH* amplification conditions, and the results demonstrated the appearance of target-specific positive lines according to the test lines designed for each marker (Figure 2A lower panel). In addition to the single primer optimization, multiplex PCR, which combined both *TLH* and *TDH* primer pairs in the same reaction, was optimized to determine the proper amplification condition for the primer pairs. Agarose gel electrophoresis results demonstrated that most of the tested annealing temperatures generated specific PCR products for both *TLH* and *TDH* genes in the same reaction. However, the *TDH* amplicon was found to be less intense when tested at 62 °C annealing temperature (Figure 2B upper panel). Similarly, when singleplex and multiplex amplicon were tested using the LFD, the visible bands were observed for both *TLH* and *TDH* test lines at all annealing conditions (Figure 2B lower panel). Therefore, based on band intensity on the agarose gel and LFD, 60 °C annealing temperature was selected.

### 3.2. Specificity Evaluation of Multiplex PCR-LFD Assay

**Figure 3:**
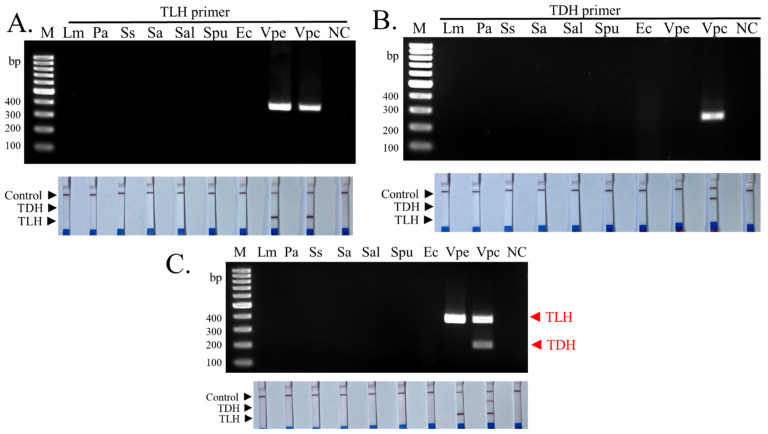
The specificity test of single primers. (**A**) The specificity of the *TLH* primer pairs against seven foodborne pathogenic bacteria visualized by agarose gel electrophoresis and LFD assay. (**B**) The specificity of the *TDH* primer pairs against seven foodborne pathogenic bacteria visualized by agarose gel electrophoresis and LFD assay. (**C**) The specificity of the multiplex PCR (*TLH* + *TDH* primers) against seven foodborne pathogenic bacteria was visualized by agarose gel electrophoresis and LFD assay. The experiments were done in duplicate. Lm: *Listeria monocytogenes* ATCC 15313, Pa: *Pseudomonas aeruginosa* ATCC 27853, Ss: *Shigella sonnei* PSU.SCB.16S.14, Sa: *Staphylococcus aureus* ATCC 25923, Sal: *Shewanella* sp. TBRC 5775, Spu: *Shewanella putrefaciens* JCM 20190, Ec: *Escherichia coli* ATCC 25922, Vpe: *V. parahaemolyticus* environmental strain, Vpc: *V. parahaemolyticus* clinical strain. NC: negative control.

To evaluate the specificity of the standardized method, strains belonging to the seven pathogenic bacteria species were used, and the results of agarose gel electrophoresis and PCR-LFD assay were compared. The results using a single primer demonstrated good PCR specificity for both *TLH* and *TDH* primer pairs. *TLH* primer pair generated positive results for the *TLH*^+^ environmental and *TDH^−^ V. parahaemolyticus* strains on agarose gel and LFD. Similarly, the *TDH* primer pair generated a specific band on the agarose gel and LFD for the *TDH*^+^ *V. parahaemolyticus* strains (Figure 3A,B upper panels). The multiplex PCR reaction demonstrated robust assay performance when *TLH* and *TDH* primer pairs were simultaneously tested. Both primer pairs did not influence each other’s amplification efficiency and specificity (Figure 3C upper panel). PCR amplicons from the multiplex PCR reaction generated expected results when tested LFD and matched with the agarose gel results (Figure 3C lower panel). These results based on the strains tested indicate the applicability of *TLH* and *TDH* genes and primer pairs for the detection and discrimination of *TDH*^+^ *V. parahaemolyticus* strains. 

### 3.3. Sensitivity Determination of Multiplex PCR-LFD Assay

**Figure 4:**
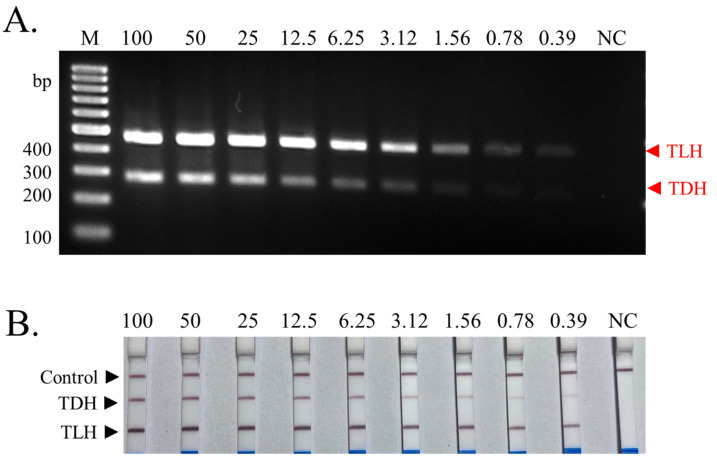
Sensitivity test of the multiplex PCR-LFD assay for the identification of pathogenic *V. parahaemolyticus.* (**A**) The results visualized by agarose gel electrophoresis. (**B**) The results visualized by LFD assay. All concentrations of DNA used are nanograms (ng)/reaction. The experiments were done in duplicate. Lane M represents 100 bp DNA ladders. NC: negative control.

The determination of the limit of detection of the multiplex PCR and LFD assay was performed using different DNA concentrations of pure culture *V. parahaemolyticus* strains (*TDH*^+^*TLH*^+^). In multiplex PCR, *TLH* amplicon was found to be positive at all tested concentrations. PCR products of *TDH* primer pairs showed the positive result in most concentrations but seemed to be fade when DNA concentrations were between 0.78 and 0.39 ng/reaction (Figure 4A). Interestingly, all concentrations of the DNA samples provided detectable test lines for both *TLH* and *TDH* amplicons in the LFD assay although the *TDH* test line displayed a slightly weak signal at 0.39 ng/reaction of the DNA sample (Figure 4B). This indicated that the multiplex PCR-LFD technique exhibited higher sensitivity, compared to conventional multiplex PCR-agarose gel electrophoresis with the limit of detection around 0.39 ng of pure culture genomic DNA sample.

### 3.4. Detection of Pathogenic TDH^+^ V. parahaemolyticus-Contaminated Samples Using Multiplex PCR-LFD Assay

**Figure 5:**
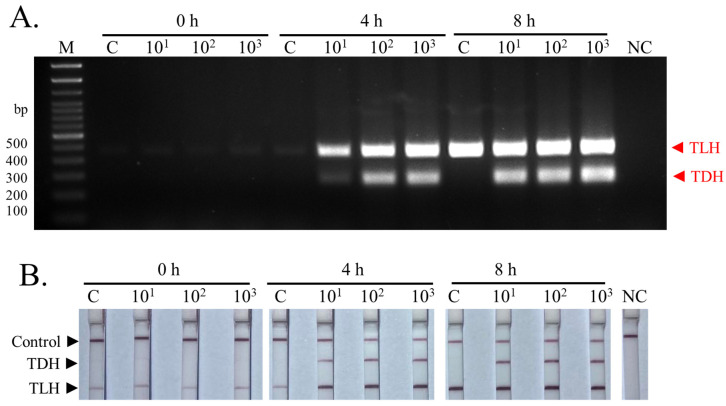
Limit of detection in spiked shrimp samples. The image shows the detection results of the multiplex PCR-LFD assay for spiked shrimp samples. The results were visualized by agarose gel electrophoresis (**A**) and LFD assay (**B**). The experiments were done in duplicate. Lane M represents 100 bp DNA ladders. NC: negative control.

In addition to the sensitivity in terms of DNA concentrations, the assay performance was evaluated using laboratory-inoculated shrimp samples. Application of the PCR products on the dipsticks demonstrated that the developed technique could detect the *TLH* gene at all times of enrichment (0, 4, and 8 h) even though the positive *TLH* amplicons were found as a faint band in samples collected at the time point of zero (Figure 5A,B). The obvious visible double detectable bands (*TLH*^+^*TDH*^+^) were found after 4 h of enrichment in all spiked samples, while no *TDH* signal was observed at these time points in uninoculated control samples. The result indicated that no pathogenic *V. parahaemolyticus* was presented as the background bacteria in the frozen shrimp. Therefore, the sensitivity of the multiplex PCR-LFD assay was 10 CFU/10 g of spiked shrimp following a 4 h enrichment period. 

## 4. Discussion

The prevalence of *V. parahaemolyticus* is the highest among 12 pathogenic *Vibrio* spp. especially in Asian countries [24,25]. The marine environment is a natural habitat for this bacterium. Therefore, seafood is the main source of contamination or outbreak of pathogenic *V. parahaemolyticus*, which has a negative impact on the seafood sector [26]. However, since not all strains of *V. parahaemolyticus* are pathogenic, the tools that can discriminate pathogenic *V. parahaemolyticus* from non-pathogenic or environmental *V. parahaemolyticus* strains are needed. In this study, the multiplex PCR method in combination with the LFD was used for the detection of pathogenic *V. parahaemolyticus* strains. The PCR-based method for pathogen detection is a stabilized method and is known for its specificity towards its target, sensitivity, and shorter turnaround time and is extensively used for the detection of foodborne pathogens, such as *Escherichia coli* [27], *Salmonella* spp. [28], and *Shigella flexneri* [29]. Moreover, the extended multiplexing method can be employed to detect multiple targets with high efficiency and flexibility. For example, Molina et al. [30] applied multiplex PCR to identify *E. coli* by targeting the *LacZ* and *yaiO* genes. Chin et al. (2017) succeeded in developing a multiplex direct PCR that could identify the serotypes of *Salmonella* directly from food samples with the relative accuracy, sensitivity, and specificity of 98.8%; 97.6%, and 100%, respectively [31].

The developed multiplex PCR-LFD assay could be used to detect pathogenic *V. parahaemolyticus*. The advantage of this assay compared to other techniques was the ability to discriminate *TDH*^+^ and *TDH*^−^ in *V. parahaemolyticus*. Furthermore, the applicability of multiplex PCR in combination with lateral flow dipstick could shorten the time for testing. Therefore, it could be potentially used as a tool for the specific detection of pathogenic *V. parahaemolyticus* strains. As a consequence, the surveillance and safety assurance of seafoods for consumers could be achieved. In this study, the primer pairs used did not display the loss of amplification efficiency according to the optimization results. It is important to optimize the annealing condition to avoid any non-specific amplification and the low amplification efficiency scenario due to the interaction of two primer pairs in the multiplex PCR reaction [32,33]. Moreover, this PCR-LFD approach has been previously employed by other studies for a similar purpose. For example, Phuakrod et al. [34] combined miniPCR and LFD assay to surpass the gel electrophoresis and imaging steps in diagnosing lymphatic filariae infection. This approach has been applied to identify or authenticate food products in terms of food mislabeling, substitution, and fraud, such as the authentication of pork [35], fish [36], and shrimp [37], thus making this approach best suited for the onsite identification of the target in a resource-limited setting [37]. 

To our knowledge, this is the first study using *TLH* and *TDH* genes-based multiplex PCR-LFD assay for detecting *V. parahaemolyticus* in seafood. The *TLH* gene was selected as *V. parahaemolyticus* species-specific marker. Data from previous published studies have demonstrated that the *TLH* gene is conserved in all *V. parahaemolyticus* strains and has been suggested as a good target for the detection of all *V. parahaemolyticus* strains [38,39,40]. For the identification of pathogenic *V. parahaemolyticus* strains, toxR [38], 16–23s rRNA [38], or bla_CARB-17_ [41] have been previously used. However, the *TDH* gene is commonly reported among pathogenic strains and has been recommended as a robust target for the identification of pathogenic *V. parahaemolyticus* strains. The World Health Organization (WHO) reported that more than 80% of pathogenic *V. parahaemolyticus* isolated from clinical patients carried this gene in their genome [20]. Additionally, studies from China and Thailand demonstrated that >90% of pathogenic *V. parahaemolyticus* harbored the *TDH* gene and used this hemolysin as a virulent factor [9,42,43]. In the present study, the developed multiplex PCR-LFD assay targeting *TLH* and *TDH* was designed in accordance with the previous reports that employed these markers for the identification of pathogenic *V. parahaemolyticus* strains. Federici et al. [14] used primers specific to *TLH* and *TDH* genes for the detection of pathogenic *V. parahaemolyticus* in clams via multiplex real-time PCR method. Another work conducted by Niu et al. also developed an assay for identifying pathogenic *V. parahaemolyticus* contamination in shrimp by targeting *TLH*, *TDH*, and ureR genes [44]. Moreover, Cheng et al. combined PCR technique and DNAzyme technology to detect *V. parahaemolyticus* with the naked eye using *TLH*, *TDH*, *TRH*, and toxR virulence genes as targets. These published studies confirmed the profound role of *TLH* and *TDH* genes as suitable markers for identifying pathogenic *V. parahaemolyticus* strains.

Different sensitivities of the PCR technique and its modified methods have been reported. Although the results of the limit of detection from the current study showed the limit of detection of the *TLH*^+^*TDH^+^* double positive of 0.78 ng (780 pg) visualized by agarose gel electrophoresis, the limit of detection was increased to 0.39 ng (390 pg) when the LFD assay was applied and was comparable to other works. For example, Hossain et al. [45] demonstrated the detection limits of developed multiplex PCR targeting groEL, *TDH*, and *TRH* genes of *V. parahaemolyticus* at 200 pg [45]. Ward and Bej [46] deployed multiplex real-time PCR with TaqMan probes to identify the total and pathogenic *V. parahaemolyticus* using *TLH*, *TDH*, *TRH*, and ORF8 genes as targets. The combined probes displayed the sensitivity of 200 ng/reaction of *V. parahaemolyticus* DNA. However, another multiplex real-time developed by Chen et al. [47] could detect the *V. parahaemolyticus* genome with a minimum detection limit of 1.4 pg/reaction using EvaGreen fluorescent dye combined with melting curve analysis. The different limits of the detection may be due to the difference in primer amplification efficiencies, reaction volume, amplicon size, master mix, and visualization methods [48,49]. A higher number of amplification cycles can be used to further improve the sensitivity of the developed method, since the number of cycles can be up to 35, which is still in the optimal range [50,51].

For the artificial spiking study, the performance and applicability of the developed multiplex PCR-LFD assay were satisfactory. This method could simultaneously detect the *TLH* and *TDH* genes after 4 and 8 h of enrichment when inoculated with 10^1^ CFU of pathogenic *V. parahaemolyticus* in 10 g of shrimp samples. Moreover, the result could discriminate pathogenic *V. parahaemolyticus* from environmental background *V. parahaemolyticus* found in frozen shrimp products, indicating the high specificity and sensitivity of the established method. Most importantly, application of a shorter enrichment time to 4 h could enable same-day detection of samples contaminated with the pathogenic *V. parahaemolyticus* strains [14]. These results are comparable to other works that require an enrichment period of approximately 3–8 h [14,15,52]. Overall, the development of multiplex PCR-LFD assay to detect and differentiate pathogenic *V. parahaemolyticus* by targeting *TLH* and *TDH* genes can be used as a tool for the identification of in the seafood sector.

## Figures and Tables

**Table 1:** Primers selected for the detection of virulence genes of *V. parahaemolyticus*.

Primer		Sequence (5′-3′)	Amplicon Size (bp)	Reference
*TLH*	F	AAA GCG GAT TAT GCA GAA GCA CTG	450	[23]
	R	GCT ACT TTC TAG CAT TTT CTC TGC		
*TDH*	F	CCA TCT GTC CCT TTT CCT GCC	269	[23]
	R	CCA CTA CCA CTC TCA TAT GC		

## Data Availability

The data presented in this study are available on request from the corresponding author.

## References

[1] Broberg C.A., Calder T.J., Orth K.V. (2011). *parahaemolyticus* Cell Biology and Pathogenicity Determinants. Microbes Infect..

[2] Yeung P.S.M., Boor K.J. (2004). Epidemiology, Pathogenesis, and Prevention of Foodborne *V. parahaemolyticus* Infections. Foodborne Pathog. Dis..

[3] Ellett A.N., Rosales D., Jacobs J.M., Paranjpye R., Parveen S. (2022). Growth Rates of *V. parahaemolyticus* Sequence Type 36 Strains in Live Oysters and in Culture Medium. Microbiol. Spectr..

[4] Baker-Austin C., Trinanes J., Gonzalez-Escalona N., Martinez-Urtaza J. (2017). Non-Cholera *Vibrios*: The Microbial Barometer of Climate Change. Trends Microbiol..

[5] Wang R., Zhong Y., Gu X., Yuan J., Saeed A.F., Wang S. (2015). The Pathogenesis, Detection, and Prevention of *V. parahaemolyticus*. Front. Microbiol..

[6] Li L., Meng H., Gu D., Li Y., Jia M. (2019). Molecular Mechanisms of *V. parahaemolyticus* Pathogenesis. Microbiol. Res..

[7] Theethakaew C., Feil E.J., Castillo-Ramírez S., Aanensen D.M., Suthienkul O., Neil D.M., Davies R.L. (2013). Genetic Relationships of *V. parahaemolyticus* Isolates from Clinical, Human Carrier, and Environmental Sources in Thailand, Determined by Multilocus Sequence Analysis. Appl. Environ. Microbiol..

[8] Yingkajorn M., Mitraparp-arthorn P., Nuanualsuwan S., Poomwised R., Kongchuay N., Khamhaeng N., Vuddhakul V. (2014). Prevalence and Quantification of Pathogenic *V. parahaemolyticus* during Shrimp Culture in Thailand. Dis. Aquat. Org..

[9] Thongjun J., Mittraparp-arthorn P., Yingkajorn M., Kongreung J., Nishibuchi M., Vuddhakul V. (2013). The Trend of *V. parahaemolyticus* Infections in Southern Thailand from 2006 to 2010. Trop. Med. Health.

[10] Tran T.H.T., Yanagawa H., Nguyen K.T., Hara-Kudo Y., Taniguchi T., Hayashidani H. (2018). Prevalence of *V. parahaemolyticus* in Seafood and Water Environment in the Mekong Delta, Vietnam. J. Vet. Med. Sci..

[11] Raghunath P. (2014). Roles of Thermostable Direct Hemolysin (*TDH*) and *TDH*-Related Hemolysin (*TRH*) in *V. parahaemolyticus*. Front. Microbiol..

[12] Kulkarni M.B., Ayachit N.H., Aminabhavi T.M. (2023). Recent Advances in Microfluidics-Based Electrochemical Sensors for Foodborne Pathogen Detection. Biosensors.

[13] Janik-Karpinska E., Ceremuga M., Niemcewicz M., Podogrocki M., Stela M., Cichon N., Bijak M. (2022). Immunosensors—The Future of Pathogen Real-Time Detection. Sensors.

[14] Federici S., Serrazanetti D.I., Guerzoni M.E., Campana R., Ciandrini E., Baffone W., Gianotti A. (2018). Development of a Rapid PCR Protocol to Detect *V. parahaemolyticus* in Clams. J. Food Sci. Technol..

[15] Park J.Y., Jeon S., Kim J.Y., Park M., Kim S. (2013). Multiplex Real-Time Polymerase Chain Reaction Assays for Simultaneous Detection of *V. cholerae*, *V. parahaemolyticus*, and *V. vulnificus*. Osong Public Health Res. Perspect..

[16] Yu J., Xing J., Zhan X., Yang Z., Qi J., Wei Y., Liu Y. (2020). Improvement of Loop-Mediated Isothermal Amplification Combined with Chromatographic Flow Dipstick Assay for *Salmonella* in Food Samples. Food Anal. Methods.

[17] Allgöwer S.M., Hartmann C.A., Holzhauser T. (2020). The Development of Highly Specific and Sensitive Primers for the Detection of Potentially Allergenic Soybean (Glycine Max) Using Loop-Mediated Isothermal Amplification Combined with Lateral Flow Dipstick (LAMP-LFD). Foods.

[18] Dai T., Yang X., Hu T., Jiao B., Xu Y., Zheng X., Shen D. (2019). Comparative Evaluation of a Novel Recombinase Polymerase Amplification-Lateral Flow Dipstick (RPA-LFD) Assay, LAMP, Conventional PCR, and Leaf-Disc Baiting Methods for Detection of *Phytophthora sojae*. Front. Microbiol..

[19] Bej A.K., Patterson D.P., Brasher C.W., Vickery M.C., Jones D.D., Kaysner C.A. (1999). Detection of Total and Hemolysin-Producing *V. parahaemolyticus* in Shellfish Using Multiplex PCR Amplification of *Tl*, *TDH* and *TRH*. J. Microbiol. Methods.

[20] World Health Organization, Food and Agriculture Organization of the United Nations (2011). Risk Assessment of V. parahaemolyticus in Seafood: Interpretative Summary and Technical Report.

[21] Wang P., Liao L., Ma C., Zhang X., Yu J., Yi L., Liu X., Shen H., Gao S., Lu Q. (2021). Duplex On-Site Detection of *V. cholerae* and *V. vulnificus* by Recombinase Polymerase Amplification and Three-Segment Lateral Flow Strips. Biosensors.

[22] Palamae S., Mittal A., Yingkajorn M., Saetang J., Buatong J., Tyagi A., Singh P., Benjakul S.V. (2022). *parahaemolyticus* Isolates from Asian Green Mussel: Molecular Characteristics, Virulence and Their Inhibition by Chitooligosaccharide-Tea Polyphenol Conjugates. Foods.

[23] Kaysner C.A., DePaola A., Jones J. (2020). BAM Chapter 9: Vibrio.

[24] Kang C.-H., Shin Y., Kim W., Kim Y., Song K., Oh E.-G., Kim S., Yu H., So J.-S. (2016). Prevalence and Antimicrobial Susceptibility of *V. parahaemolyticus* Isolated from Oysters in Korea. Environ. Sci. Pollut. Res. Int..

[25] Law J.W.-F., Ab Mutalib N.-S., Chan K.-G., Lee L.-H. (2014). Rapid Methods for the Detection of Foodborne Bacterial Pathogens: Principles, Applications, Advantages and Limitations. Front. Microbiol..

[26] Gavilan R.G., Caro-Castro J., Blondel C.J., Martinez-Urtaza J. (2023). *Vibrio parahaemolyticus* Epidemiology and Pathogenesis: Novel Insights on an Emerging Foodborne Pathogen. Adv. Exp. Med. Biol..

[27] Hariri S. (2022). Detection of *Escherichia coli* in Food Samples Using Culture and Polymerase Chain Reaction Methods. Cureus.

[28] Awang M.S., Bustami Y., Hamzah H.H., Zambry N.S., Najib M.A., Khalid M.F., Aziah I., Abd Manaf A. (2021). Advancement in *Salmonella* Detection Methods: From Conventional to Electrochemical-Based Sensing Detection. Biosensors.

[29] Brengi S.P., Sun Q., Bolaños H., Duarte F., Jenkins C., Pichel M., Shahnaij M., Sowers E.G., Strockbine N., Talukder K.A. (2019). PCR-Based Method for *Shigella flexneri* Serotyping: International Multicenter Validation. J. Clin. Microbiol..

[30] Molina F., López-Acedo E., Tabla R., Roa I., Gómez A., Rebollo J.E. (2015). Improved Detection of *Escherichia coli* and Coliform Bacteria by Multiplex PCR. BMC Biotechnol..

[31] Chin W.H., Sun Y., Høgberg J., Quyen T.L., Engelsmann P., Wolff A., Bang D.D. (2017). Direct PCR—A Rapid Method for Multiplexed Detection of Different Serotypes of *Salmonella* in Enriched Pork Meat Samples. Mol. Cell. Probes.

[32] Zhang M., Wu J., Shi Z., Cao A., Fang W., Yan D., Wang Q., Li Y. (2022). Molecular Methods for Identification and Quantification of Foodborne Pathogens. Molecules.

[33] Mukhopadhyay A., Mukhopadhyay U.K. (2007). Novel Multiplex PCR Approaches for the Simultaneous Detection of Human Pathogens: *Escherichia coli* 0157:H7 and *Listeria monocytogenes*. J. Microbiol. Methods.

[34] Phuakrod A., Sripumkhai W., Jeamsaksiri W., Pattamang P., Loymek S., Brindley P.J., Sarasombath P.T., Wongkamchai S. (2021). A MiniPCR-Duplex Lateral Flow Dipstick Platform for Rapid and Visual Diagnosis of Lymphatic Filariae Infection. Diagnostics.

[35] Yin R., Sun Y., Wang K., Feng N., Zhang H., Xiao M. (2020). Development of a PCR-Based Lateral Flow Strip Assay for the Simple, Rapid, and Accurate Detection of Pork in Meat and Meat Products. Food Chem..

[36] Taboada L., Sánchez A., Pérez-Martín R.I., Sotelo C.G. (2017). A New Method for the Rapid Detection of Atlantic Cod (*Gadus morhua*), Pacific Cod (*Gadus macrocephalus*), Alaska Pollock (*Gadus chalcogrammus*) and Ling (*Molva molva*) Using a Lateral Flow Dipstick Assay. Food Chem..

[37] Kwawukume S., Velez F.J., Williams D., Cui L., Singh P. (2023). Rapid PCR-Lateral Flow Assay for the Onsite Detection of Atlantic White Shrimp. Food Chem..

[38] Xing J., Yu J., Liu Y. (2020). Improvement and Evaluation of Loop-Mediated Isothermal Amplification Combined with Chromatographic Flow Dipstick Assays for *V. parahaemolyticus*. J. Microbiol. Methods.

[39] Nordstrom J.L., Vickery M.C.L., Blackstone G.M., Murray S.L., DePaola A. (2007). Development of a Multiplex Real-Time PCR Assay with an Internal Amplification Control for the Detection of Total and Pathogenic *V. parahaemolyticus* Bacteria in Oysters. Appl. Environ. Microbiol..

[40] Letchumanan V., Chan K.-G., Lee L.-H. (2014). *V. parahaemolyticus*: A Review on the Pathogenesis, Prevalence, and Advance Molecular Identification Techniques. Front. Microbiol..

[41] Hu Y., Huang X., Guo L., Shen Z., LV L., Li F., Zhou Z., Zhang D. (2021). Rapid and Visual Detection of *V. parahaemolyticus* in Aquatic Foods Using Bla_CARB-17_ Gene-Based Loop-Mediated Isothermal Amplification with Lateral Flow Dipstick (LAMP-LFD). J. Microbiol. Biotechnol..

[42] Chen X., Zhu Q., Liu Y., Wang R., Xie H., Chen J., Cheng Y., Zhang H., Cao L., Chen Y. (2020). Pathogenic Characteristics of and Variation in *V. parahaemolyticus* Isolated from Acute Diarrhoeal Patients in Southeastern China from 2013 to 2017. Infect. Drug Resist..

[43] Chen Y., Chen X., Yu F., Wu M., Wang R., Zheng S., Han D., Yang Q., Kong H., Zhou F. (2016). Serology, Virulence, Antimicrobial Susceptibility and Molecular Characteristics of Clinical *V. parahaemolyticus* Strains Circulating in Southeastern China from 2009 to 2013. Clin. Microbiol. Infect..

[44] Niu B., Hong B., Zhang Z., Mu L., Malakar P.K., Liu H., Pan Y., Zhao Y. (2018). A Novel QPCR Method for Simultaneous Detection and Quantification of Viable Pathogenic and Non-Pathogenic *V. parahaemolyticus* (*Tlh*^+^, *TDH*^+^, and UreR^+^). Front. Microbiol..

[45] Hossain M.T., Kim Y.-O., Kong I.-S. (2013). Multiplex PCR for the Detection and Differentiation of *V. parahaemolyticus* Strains Using the GroEL, *TDH* and *TRH* Genes. Mol. Cell. Probes.

[46] Ward L.N., Bej A.K. (2006). Detection of *V. parahaemolyticus* in Shellfish by Use of Multiplexed Real-Time PCR with TaqMan Fluorescent Probes. Appl. Environ. Microbiol..

[47] He P., Chen Z., Luo J., Wang H., Yan Y., Chen L., Gao W. (2014). Multiplex Real-Time PCR Assay for Detection of Pathogenic *V. parahaemolyticus* Strains. Mol. Cell. Probes.

[48] Wei S., Zhao H., Xian Y., Hussain M.A., Wu X. (2014). Multiplex PCR Assays for the Detection of *V. alginolyticus*, *V. parahaemolyticus*, *V. vulnificus*, and *V. cholerae* with an Internal Amplification Control. Diagn. Microbiol. Infect. Dis..

[49] Sea-liang N., Sereemaspun A., Patarakul K., Gaywee J., Rodkvamtook W., Srisawat N., Wacharaplusadee S., Hemachudha T. (2019). Development of Multiplex PCR for Neglected Infectious Diseases. PLoS Negl. Trop. Dis..

[50] Lorenz T.C. (2012). Polymerase Chain Reaction: Basic Protocol Plus Troubleshooting and Optimization Strategies. J. Vis. Exp..

[51] Sabat G., Rose P., Hickey W.J., Harkin J.M. (2000). Selective and Sensitive Method for PCR Amplification of *Escherichia coli* 16S rRNA Genes in Soil. Appl. Environ. Microbiol..

[52] Xu D., Ji L., Wu X., Yan W., Chen L. (2018). Detection and Differentiation of *V. parahaemolyticus* by Multiplexed Real-Time PCR. Can. J. Microbiol..

